# Efficacy of *Annona squamosa* L in the Synthesis of Glycosaminoglycans and Collagen during Wound Repair in Streptozotocin Induced Diabetic Rats

**DOI:** 10.1155/2014/124352

**Published:** 2014-06-09

**Authors:** Thangavel Ponrasu, Lonchin Suguna

**Affiliations:** Department of Biochemistry, Central Leather Research Institute, Council of Scientific and Industrial Research (CSIR), Adyar, Chennai 600 020, India

## Abstract

The aim of this work was to find out the effects of *Annona squamosa* on the formation of glycosaminoglycans and collagen during wound healing in normal and diabetic rats. Diabetes induced rats were segregated into 4 groups, each containing six animals. Groups I and III served as the normal and diabetic control while groups II and IV served as normal and diabetic treated. The animals were treated with 200 **μ**L of *Annona squamosa* extract topically. The granulation tissues formed were removed on the 8th day and the amount of glycosaminoglycans (GAGs) and collagen formed was evaluated by sequential extraction and SDSPAGE, respectively. Histological evaluation was also carried out using Masson's trichrome stain. *In vitro* wound healing efficacy of *A. squamosa* in human dermal fibroblast culture (HDF) was also carried out. The fibroblasts treated with varying concentrations of *A. squamosa* were examined for proliferation and closure of the wound area and photographed.* A. squamosa* increased cellular proliferation in HDF culture. The granulation tissues of treated wounds showed increased levels of glycosaminoglycans (*P* < 0.05) and collagen which were also confirmed by histopathology. The results strongly substantiate the beneficial effects of *A. squamosa* on the formation of glycosaminoglycans and collagen during wound healing.

## 1. Introduction

Wound healing is the process of repair that follows injury to the skin and other soft tissues. Wound healing cascade begins at the moment of injury or insult and progresses toward complete closure of the wound. Healing requires well-orchestrated integration of the complex biological and molecular events of cell migration, proliferation, and extracellular matrix (ECM) deposition [1]. The distinct interrelated phases which play a crucial role in wound healing are hemostasis, inflammation, proliferation, and remodeling [2]; the end product of wound healing is a dense connective tissue (scar) consisting predominantly of collagen [3, 4].

Diabetic wounds are defined as chronic wounds or lesions that take long time to heal or fail to heal [5]. Chronic wounds fail to progress through a normal, orderly, and timely sequence of repair. These wounds may eventually pass through the repair process without restoring sustained anatomical and functional results [6].

Proteoglycans are believed to be involved in the regulation of collagen fibrillogenesis and cell growth and act as tissue organizers [7]. Proteoglycans are the most abundant noncollagenous molecules available in the extracellular matrix, associated with collagen fibrils to form an assembly of the fibrils* in vivo*, and they may reduce diameter of fibrils by inhibiting lateral growth of fibrils [8]. Glycosaminoglycans (GAGs) are known as collagen associated proteoglycans (PGs) in the extracellular matrix of cell membranes and connective tissues. Glycosaminoglycans and proteoglycans significantly influence the cellular proliferation and rate of wound healing [9].

Proteoglycans, an essential component of mature matrix, are actively synthesized during the proliferative phase of wound healing. Fibrin is the primary matrix at the wound site replaced by collagen and proteoglycans gradually [10].

Collagens are the major structural component of the connective tissues, composed of three identical polypeptide chains, each chain comprising Gly-X-Y, where G is glycine and X and Y are the imino acids proline and hydroxy proline, respectively. This sequence contributes to the formation of triple helical structure of collagen [11]. Both collagen and glycosaminoglycans play a key role in wound healing. Any agent which affects the normal metabolism of these important connective tissue proteins will definitely affect the normal wound healing pattern [12].

Plant extracts have been widely used as potent wound healing agents throughout the world. The role of plant extracts in glycosaminoglycans and collagen metabolism has been well documented [13, 14].

*Annona squamosa *L. (Annonaceae), commonly known as custard apple, is a native of West Indies and is cultivated throughout India, mainly for its edible fruit. Phytochemical screening and efficacy of this plant on wound healing in diabetic rats have already been reported by us [15].

In this paper, we report the influence of* A. squamosa* extract on the synthesis and characterization of glycosaminoglycans and collagen with detailed histological analysis during wound healing in streptozotocin-nicotinamide induced diabetic rats. This study also explores the role of* A. squamosa* in fibroblast proliferation with respect to wound closure rate. 

## 2. Materials and Methods

### 2.1. Plant Collection and Extraction

*A. squamosa* leaves were procured, shade-dried, and crushed to make fine powder. 100 g of this powder was macerated with 70% ethanol in dark and filtered to harvest a viscous supernatant. The extract was dried under vacuum below 40°C. The viscous residue was collected, weighed, and kept at 4°C until use. The extract was reconstituted in phosphate buffered saline (PBS) whenever required.

### 2.2. Chemicals

D-Glucuronic acid, chondroitin sulphate B, chondroitinase ABC, chondroitinase AC,* Streptomyces* hyaluronidase, *β*-D-galactosidase, sodium dodecyl sulphate, coomassie blue R-250, N,N-methylene bisacrylamide, and ß-mercaptoethanol were purchased from Sigma Chemical Company, St. Louis, USA. Pepsin, proteinase K, and potassium acetate were procured from Sisco Research Laboratory, Mumbai, India. All other reagents were of high analytical grade.

### 2.3. *In Vitro* Studies

#### 2.3.1. MTT Assay

For quantitative evaluation of cell viability and proliferation, MTT (3-[4,5-dimethylthiazol-2-yl]-2,5-diphenyl tetrazolium bromide) assay was used, in which only viable cells can reduce MTT to insoluble purple formazan [16]. Thus, the intensity of purple color represents the number of viable cells. For MTT assay, the HDF cells were cultured in 24-well microtiter plate at a density of 5 × 10^3^ cells per 100 *μ*L for 48 h. The culture medium was supplemented with different concentrations of* A. squamosa* like 7.8, 15.6, 31.2, 62.5, 125, 250, 500, and 1000 parts per million (ppm). The cells were then incubated in a humidified atmosphere of 5% CO_2_ at 37°C and medium was changed every day (wherever applicable). MTT assay was performed at 48 h after treatment. The formazan crystals formed by living cells were solubilized with DMSO and the absorbance was measured at 570 nm with background subtraction at 690 nm using multimode plate reader (TECAN, Infinite M 200).

#### 2.3.2. *In Vitro* Wound Healing Assay

The* in vitro* wound healing assay was carried out as previously described by Kheradmand et al. [17]. HDF cells were seeded on 24-well tissue culture dishes (10^6^ cells/well). The cells were incubated for 48 h at 37°C with 5% CO_2_. When confluence was reached, cell monolayers were incubated for 12 h in serum-free medium. The monolayers were then gently scratched with a sterile pipette tip and extensively rinsed with medium to remove all cellular debris. An indicated concentration of plant extract was added and incubated for 24, 48, and 72 h. The average extent of wound closure was evaluated by measuring the width of the wound. The rate of proliferation of cells into the wound area was examined by placing the cells under an inverted microscope and the image obtained was photographed.

### 2.4. *In Vivo* Studies

#### 2.4.1. Experimental Design and Diabetes Induction

Healthy male Wistar albino rats (150–200 g) were used for the* in vivo* wound healing study. The rats were fed with commercial rat feed and water* ad libitum*. All clinical procedures were carried out according to the guidelines of the Institutional Animal Care and Use Committee (IACUC). A formal approval from the Animal Ethical Committee has also been obtained.

Diabetes was induced by a single intraperitoneal injection of streptozotocin (50 mg/kg b·wt) dissolved in 0.1 M of cold citrate buffer (pH 4.5), 15 min after the intraperitoneal administration of nicotinamide (110 mg/kg b·wt) in overnight fasted rats [18, 19]. Diabetic status was confirmed by tail vein blood glucose estimation using glucometer (One Touch Horizon, Johnson & Johnson, Mumbai, India) after 72 h. Two weeks after diabetic induction, rats with blood glucose level >250 mg dL^−1^ were deemed diabetic and used for the experiment.

The rats were divided into four groups comprising six rats in each group as given below:

 group I: control rats left untreated,
 group II: rats treated with* A. squamosa* (200 *μ*L) at a concentration of (100 mg/kg b·wt),
 group III: diabetic control rats also left untreated,
 group IV: diabetic rats treated with* A. squamosa *(200 *μ*L) at a concentration (100 mg/kg b·wt).

#### 2.4.2. Excision Wound and Drug Administration

Wounds were created after the confirmation of diabetes. Rats were anaesthetized by the intraperitoneal injection of thiopentone (50 mg/kg b·wt) dissolved in sterile distilled water [20]. A 2 cm^2^ (4 cm) full thickness open excision wound was made on the back of the rat as reported in our earlier studies [15]. The control rats were left untreated and the treated rats were administered once daily with 200 *μ*L (100 mg/kg b·wt reconstituted in PBS) of the extract for 8 days. The animals were sacrificed and the wound tissues were removed on day 8 after wounding and used for the glycosaminoglycan and collagen analyses.

#### 2.4.3. Glycosaminoglycans (GAGs)

Total GAGs from the wound tissues were extracted as described by Smith et al. [21]. The amount of GAGs was determined by estimating uronic acid content [22]. Individual GAGs were estimated using GAG degrading enzymes and nitrous acid treatment as described by Breen et al. [23].

#### 2.4.4. Collagen

Fractionation of collagen was performed by the method of Piez [24]. The susceptibility of insoluble collagen to denaturing agents like urea and potassium thiocyanate was analyzed by the method of Adam et al. [25]. The aldehyde content of the acid soluble collagen was estimated according to the method of Paz et al. [26].

#### 2.4.5. SDS-PAGE

The subunit composition of the isolated collagen was investigated by sodium dodecyl sulfate-polyacrylamide gel electrophoresis (SDS-PAGE) [27]. Briefly, collagen bands were separated by SDS-PAGE using 3% stacking gel with 5% separating gel and Coomassie brilliant blue staining. Interrupted SDS-PAGE was used for the separation of type III collagen.

### 2.5. Histopathology

The rats were sacrificed and the tissues were removed from the wound site periodically. These samples were then separately fixed in 10% formalin-saline, dehydrated through graded alcohol series, cleared in xylene, and embedded in paraffin wax (melting point 56°C). Serial sections of 5 *μ*m were cut and stained with Masson's trichrome. The sections were examined under light microscope and photomicrographs were taken for the analysis.

### 2.6. Statistical Analysis

Data were expressed as mean ± SD of six animals in each group and the results were statistically evaluated using one-way ANOVA and Student's paired* t*-test. All statistical analyses were performed using GraphPad prism (version 5.0; GraphPad software Inc., San Diego, CA, USA). Values corresponding to *P* < 0.05 were considered as significant.

## 3. Results

### 3.1. Cell Viability Assay

**Figure 1:**
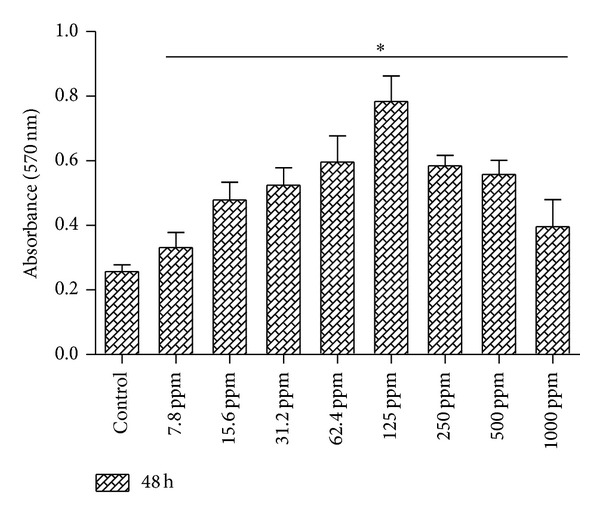
Efficacy of* A. squamosa* extract on the proliferation of HDF cells for 48 hrs. HDF cells were cultured with graded concentrations of* A. squamosa* extract (7.8 ppm–1000 ppm) and the number of viable cells was determined by MTT assay after 48 hrs. The results revealed that the* A. squamosa *extract increased the viable cells with increasing concentrations and there is no toxic or adverse effect at any concentration. All the data were expressed as mean ± SD (*n* = 6) and statistically significant (*P < 0.05) when compared to control culture.

The proliferation and viability of the cells in the presence of different concentrations of the extract (7.8–1000 ppm) were observed for 48 h, by MTT assay. A significant increase in the cell viability was observed up to a concentration of 125 ppm as depicted in Figure 1. The proliferation was found to increase within the same cells day by day (data not shown). Culture supplemented with high concentrations like 250, 500, and 1000 ppm showed slightly lesser number of viable cells than the concentrations up to 250 ppm. Based on this, concentrations between 7.8 and 125 ppm of* A. squamosa* extract were used for further study.

### 3.2. Efficacy of* A. squamosa* in* In Vitro* Wound Healing

**Figure 2:**
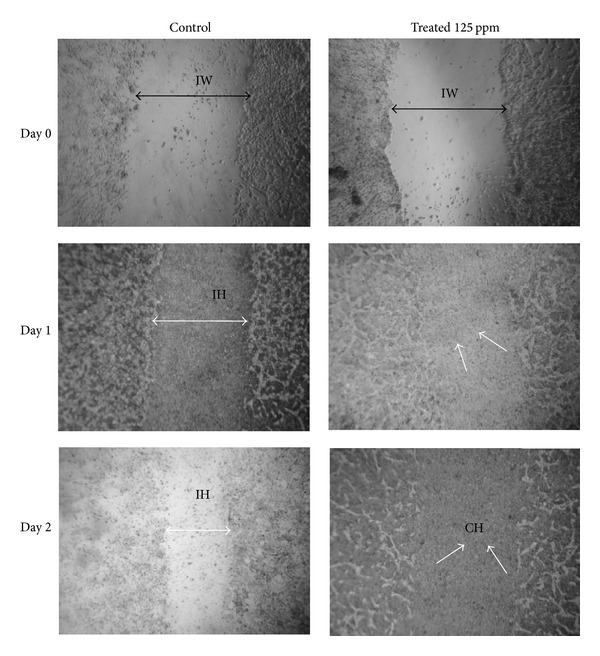
*In vitro* wound healing of HDF cells of control culture and* A. squamosa* treated (supplemented with 7.8–125 ppm) cultures. Proliferation of HDF cells was observed for 2 days after wound creation. The double headed black arrow shows the margin of scratched area on day 0 as initial wound (IW), and double headed white arrow shows the incomplete healing (IH), whereas white arrow shows the complete healing (CH).* A. squamosa* treatment shows complete healing on day 2, which clearly indicates that the* A. squamosa* accelerates the rate of proliferation faster in treated culture than the control.

*In vitro* wound healing assay was carried out using scratch wound model in confluent HDF culture. The proliferation and wound closure were evaluated until the completion of healing. After making the scratch, the cells were supplemented with 7.8–125 ppm of* A. squamosa* extract and photomicrographs were taken using inverted microscope to assess wound healing at different time point intervals.  Figure 2 shows the images of the scratch wounds treated with 125 ppm of* A. squamosa*, taken on days 0, 1, and 2, as this concentration showed the best results.* A. squamosa* supplementation showed enhanced proliferation of HDF cells and wound closure was completed within two days, whereas part of incomplete wound was observed in control culture and it took almost three days to complete healing. For each concentration and each time frame, three scratched wells were used to assess wound healing. These results suggested that* A. squamosa* has no cytotoxic effect and has significant effect on proliferation of HDF cells in* in vitro*, which would be advantageous for reepithelialization during wound healing.

### 3.3. Effect of* A. squamosa* on Glycosaminoglycans

**Figure 3:**
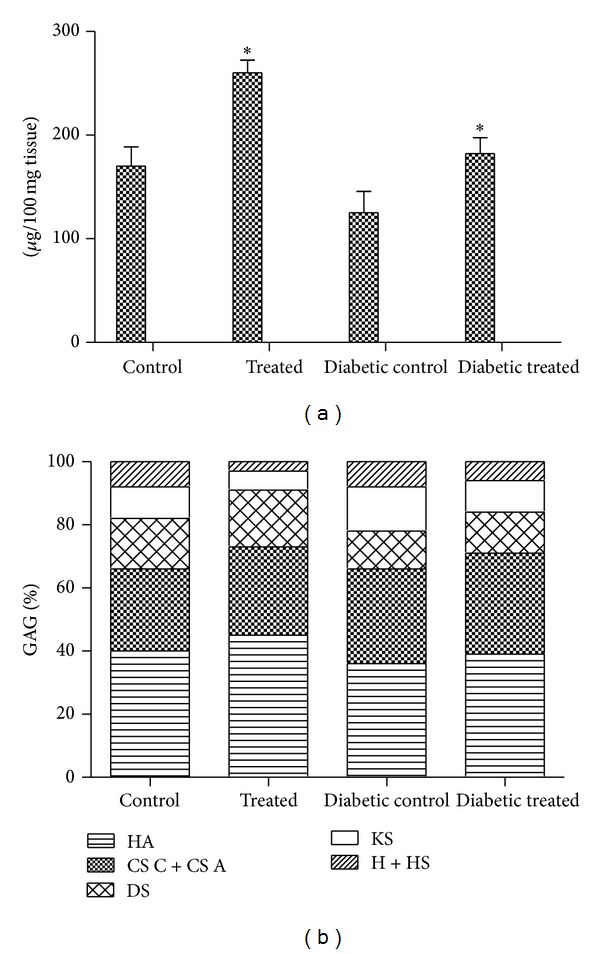
(a) Glycosaminoglycans of control and* A. squamosa* treated wound tissues on day 8 after wounding. Values are expressed as mean ± SD for six animals. *P < 0.05 is considered as significant compared with the control. (b) Percentage proportions of various glycosaminoglycans formed in control and* A. squamosa* treated wound tissues on day 8 after wounding. HA: hyaluronic acid; CS C + CS A: chondroitin sulphate A and C; DS: dermatan sulphate; KS: keratan sulphate; H + HS: heparin and heparin sulphate.

Levels of total GAGs evaluated on 8th day wound tissues of the control and treated rats are shown in Figure 3. The values are expressed as uronic acid equivalence. Synthesis of ground substances was found to be high in treated groups. The amount of total GAGs was significantly (*P* < 0.05) higher (53%) in normal treated (group II) than control. In group IV, the increase was around 46% when compared to group III diabetic control (Figure 3(a)). Percentage of individual GAGs determined in the wound tissues of control and* A. squamosa* treated groups is shown in Figure 3(b). Among the various GAGs formed in the wound tissues, hyaluronic acid levels have a major proportion in both untreated and treated groups. In normal treated group, about 11% increase was observed when compared to control. Similar trend (8%) was observed in diabetic treated group for hyaluronic acid. A 7% and a 6% increase in chondroitin sulfates were observed in normal treated and diabetic treated groups as compared to their respective controls. Dermatan sulfate was also significantly increased in normal treated group (7%) and diabetic treated group (11%) when compared to control. But, keratan sulfate content was significantly reduced in both diabetic treated (29%) and normal treated groups (40%) when compared to their respective controls.

### 3.4. Effect of* A. squamosa* on Collagen

Table 1 illustrates the solubility pattern of collagen of day 8 tissues of control and treated rats in terms of neutral salt soluble (NSS), acid soluble (AS), and pepsin soluble (PS) collagen and insoluble collagen (IS). In all groups, pepsin digestion showed a significantly higher amount of collagen particularly in* A. squamosa* treated rats. The amount of AS fraction was greatly improved in* A. squamosa* treated groups. The difference between normal and diabetic treated group was found to be 18%. A similar kind of trend was observed for IS in treated groups. It shows that there was abundant and earlier maturation of collagen fibers in* A. squamosa* treatment.

The susceptibility AS to different denaturing agents like 2 M KCNS and 6 M urea was carried out in both control and treated wound tissues. The amount of collagen released by these agents was significantly (*P* < 0.05) reduced in* A. squamosa* treated rats. 2 M KCNS treatment showed 46% release for control and 33% for extract treated AS. Similarly, 41% and 34% of decreased collagen release were obtained for both diabetic control and diabetic treated (Table 2).

*A. squamosa* treated wound tissues showed high aldehyde content in the pepsin soluble collagen. There were about 46% increase in normal and 38% increase in diabetic treated groups when compared to control (Table 3). The increase in aldehyde content and reduction of susceptibility pattern in* A. squamosa* treated rats significantly explain the formation and maturation of collagen.

### 3.5. SDS-PAGE

**Figure 4:**
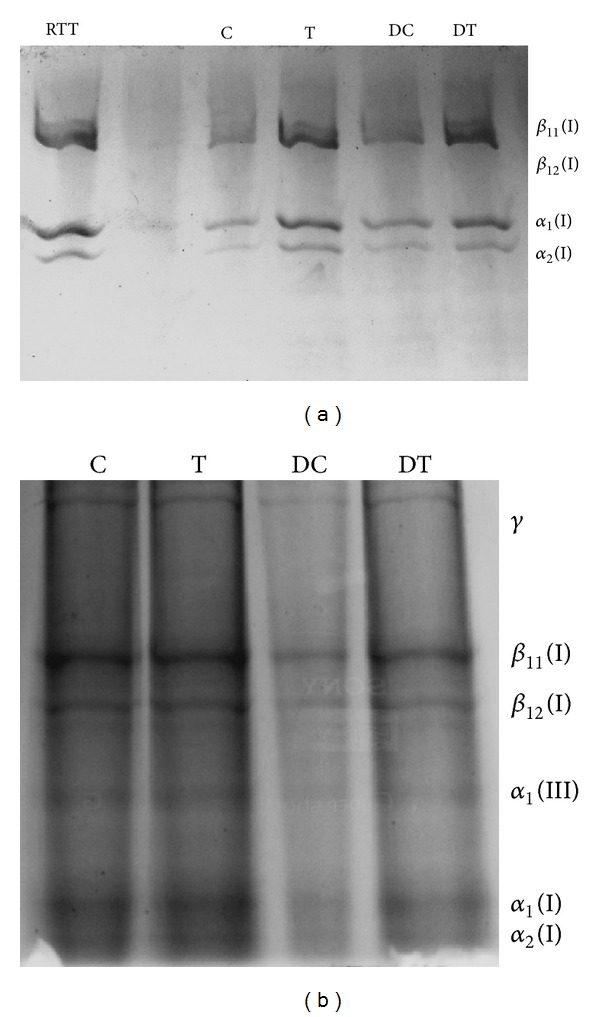
Normal (a) and interrupted (b) SDS-PAGE photograph showing the distribution of collagen. All lanes were loaded with equal concentration of collagen (25 *μ*g). RTT: rat tail tendon; C: control; T: treated; DC: diabetic control; DT: diabetic treated.

Figure 4(a) shows the SDS-PAGE pattern of pepsin soluble collagen from control and treated wound tissues. From the banding pattern, we could observe a significant increase in type I collagen in* A. squamosa* treated normal and diabetic rats.  Figure 4(b) depicts the interrupted gel electrophoresis of pepsin soluble collagen, from which we could observe a marked increase in type III collagen in the treated groups.

### 3.6. Histopathology of Wound Tissues

**Figure 5:**
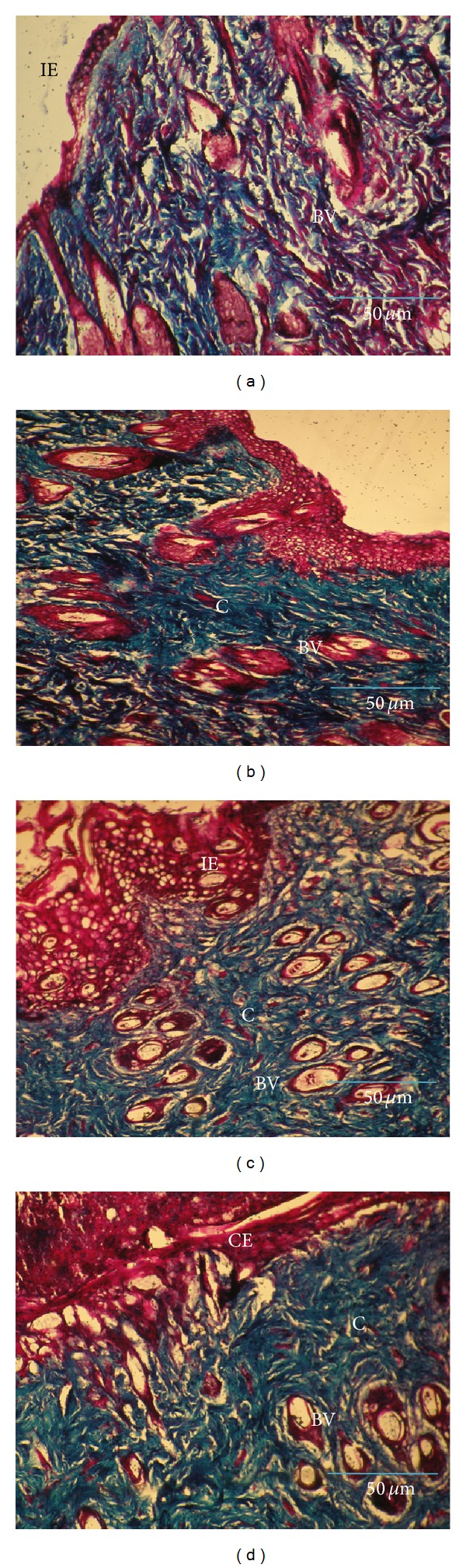
Masson's trichrome staining for collagen in control and* A. squamosa* treated wound tissues from first and second week, respectively (magnification 20x). Control (a) shows collagen fibers with incomplete epithelialization whereas (b) treated tissue shows dense collagen deposition. In the second week, control (c) depicts thin collagen layer at the wound site and (d) treated tissue shows complete epithelialization with uniform collagen deposition. IE: incomplete epithelialization, CE: complete epithelialization, E: epithelialization, BV: blood vessels, and C: collagen. Scale bar: 50 *μ*m.

A detailed histological evaluation was carried out to screen the collagen maturation using Masson's trichrome staining. Masson's trichrome stained wound tissue of the first week normal control showed less collagen fibers, proliferating blood capillaries, and incomplete epithelialization (Figure 5(a)). Whereas, complete fibrous tissue, more number of collagen bundles and large number of blood vessels were observed in treated tissue (Figure 5(b)). In the second week, the amount of collagen deposited on the wound surface in control tissue was found to be inadequate (Figure 5(c)). But, more accumulation of collagen fibers was seen in treated tissue (Figure 5(d)).

**Figure 6:**
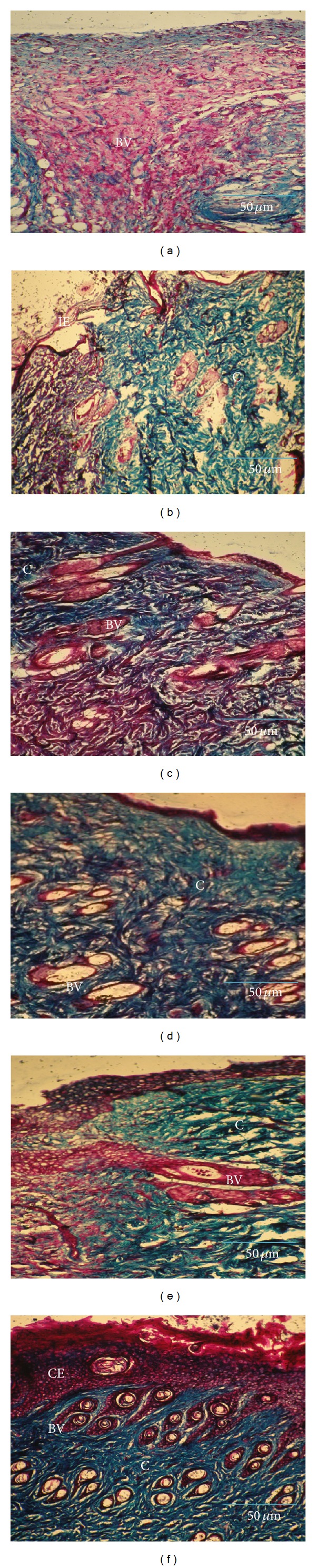
Masson's trichrome staining for collagen in control and* A. squamosa* treated diabetic wound tissues from first, second, and third week, respectively (magnification 20x). Control (a) shows large number of proliferating blood vessels, whereas (b) treated tissue shows less collagen formation. In the second week, control (c) depicts adequate amount of blood vessels formation and collagen fibers and (d) treated tissue shows thick collagen fiber deposition under thin epithelial layer. In the third week, control (e) shows accumulation of loosely arranged collagen bundles at the wound site and (f) explores the completed epithelial layer with highly organized collagen deposition. IE: incomplete epithelialization, E: epithelialization, C: collagen, and BV: blood vessels. Scale bar: 50 *μ*m.

In diabetic group, the first week of control tissue showed large number of blood vessels with minimal cellular infiltrates on the wounded site (Figure 6(a)). In treated tissue, large amount of collagen has been observed with an early epithelialization process (Figure 6(b)). The second week of control tissue showed equal amount of blood capillaries and collagen bundles at the wound site (Figure 6(c)). Thick collagen fiber deposition under the completed epithelial layer on the wound surface was seen in treated tissue (Figure 6(d)). In the third week, fewer amounts of loosely packed collagen molecules were accumulated in untreated tissue depicting incomplete wound closure (Figure 6(e)). But, highly organized thick collagen fibers as bundles and thick uniform epithelial layer were formed in treated tissue (Figure 6(f)).

## 4. Discussion

In this investigation, efficacy of* A. squamosa* extract in* in vitro* cell viability, proliferation of HDF cells, and its wound closure was studied to confirm the therapeutic activity of this extract in wound healing.

Even though various models have been proposed to study reepithelialization process in* in vitro*, the simple one is fibroblast monolayer scratch wound, in which injury was mechanically created in confluent fibroblast culture using sterile microtip and recovery of wound area has been used as an indicator for wound reepithelialization [28]. After scratch wounding, the neighboring intact mesenchymal cells start to migrate rapidly over the wounded area until they cover the wound surface with cells. Then the cells begin to proliferate to increase number of viable cells that restore the normal epithelial structure. Later, the fibroblasts start to differentiate on the wounded surface area of the healed site and become soft and smooth, and a well-layered architecture is restored [29]. In our results, we found that all the concentrations of* A. squamosa* extract (7.8 ppm–1000 ppm) increased the number of viable cells and proliferation of fibroblasts and cover the wound surface rapidly. There is no significant toxicity and adequate antiproliferative effect observed in* A. squamosa* treatment.

Cell proliferation is an essential event during reepithelialization, so proliferating fibroblasts at the wound site ensure an adequate supply of cells to migrate and cover the wound surface. Synthesis of extracellular matrix (ECM) is a key feature of wound healing. Dermal reconstruction is characterized by the formation of granulation tissue, which includes cell proliferation, ECM deposition, wound contraction, and angiogenesis [30].

After tissue injury, synthesis of ground substances plays an important role during wound healing process. They are mainly PGs and GAGs. GAGs have been found to be regulators of cellular proliferation, migration, and differentiation [31]. Synthesis of these substances and their degradation has great impact on the healing process. The GAGs are the first components of the extracellular matrix to be synthesized during wound healing and form the scaffold for collagen and elastin deposition [32]. Our results revealed that* A. squamosa* extract significantly improved the total GAGs in normal and diabetic treated rats more than the control.

HA, a known glycosaminoglycan, is an important physiological substance that plays a vital role in the healthy formation of connective tissue [33]. HA has also been shown to stimulate collagen synthesis in fetal fibroblast cultures [34]. Fibroblasts are the major components which secrete hyaluronic acid into the extracellular matrix [35]. The role of hyaluronate gel in wound healing in streptozotocin induced diabetic rats has already been reported [36].

HA might be the rationale behind the availability of excess fluid and flexible matrix on the wound site that facilitates greater cell mobility and easier and faster regeneration of damaged tissue. The increased content of HA in* A. squamosa *treated wounds may result in the formation of a more stable scar [13]. Chondroitin sulfates A and C have a crucial role in* in vitro* proliferation of fibroblasts to accelerate the wound closure rapidly through their location of sulfation group [37]. Dermatan sulphate proteoglycans are closely associated with collagen fibers [38]. They influence the collagen fibril formation* in vitro* and may therefore contribute to the organization and strength of the collagen fibrillar assembly in wound tissues [39].

A significant reduction in the levels of keratan sulfate in the treated groups was observed. Funderburgh has reported that myofibroblasts exhibited reduced expression of keratocan, a keratan sulfate linked proteoglycan. The increased rate of contraction in the treated groups suggested that* A. squamosa* extract accelerates the transformation of fibroblasts to myofibroblasts. Reduced level of keratan sulfate in the wound tissue is associated with inflammation, suggesting the role of proinflammatory cytokines involved in the downregulation of keratan sulfate biosynthesis [40, 41]. Cintron et al. have shown that corneal wound healing resulted in a reduction of keratan sulfate and in accumulation of highly sulfated chondroitin/dermatan sulfate in the scar. The results we obtained also substantiate this observation [42].

Collagen, a principal component of connective tissue, plays a major role in the healing of wounds by providing a structural architecture for the remodeling tissue. Collagen molecules contain aldehydic groups that cross-link with amino acids to form collagen fibers. Normally, an increase in collagen synthesis would result in an increase in aldehyde content which leads to a greater potential for cross-linking [43, 44].

The present investigation shows that the collagen obtained from* A. squamosa *treated wounds has a higher content of aldehydic groups (Table 3) than collagen from controls. This observation indicates that the collagen in treated wounds must have undergone a greater degree of cross-linking resulting in an ultimate increase in wound strength, which was further confirmed by the tensile strength of the wounds.

Solubility pattern of collagen in neutral salt buffer and acid solution mainly depends on the cross-linking of collagen. Highly cross-linked collagen becomes less soluble in neutral salt buffer and acid solution which can be released only in pepsin digestion.* A. squamosa *treatment showed decreased percentage of solubility in neutral buffer and in dilute acid solution. A significantly larger amount was solubilized in pepsin digestion and this is an indication of increased levels of cross-linking in treated groups. The insoluble collagen content of treated groups is also higher than that of the control group.

Type I collagen is the most abundant type of collagen observed in normal dermis (approximately 80% to 90%). But during the early phases of wound healing, fibroblasts actively produce type III collagen, which may account for 30% of the total collagen in a healing wound. By week 2, type I collagen again becomes the principal collagen produced by fibroblasts. Type I and type III collagen are formed in skin in a higher proportion relative to other types and are maintained in a fixed proportion relative to one another in normal skin tissue [45].* A. squamosa *treated wounds synthesize greater amounts of type III collagen when compared to controls. The presence of higher levels of type III collagen may have beneficial effects on early wound healing process and result in better organization of type I collagen in the final scar [32].

Histological evaluations using specific stains strongly support these results. A greater degree of epithelialization and collagen deposition observed in* A. squamosa* treated wounds reveals the healing efficacy of the plant extract. Proliferated blood capillaries and dense collagen fibers were uniformly distributed, significantly increased at the wound site in normal treated and diabetic treated rats, especially during second and third week, respectively. These changes indicate a shift in wound healing from the proliferative phase to the maturation phase.

Earlier, we have reported that the rate of wound contraction was significantly higher and period of epithelialization was shorter in normal and diabetic treated rats, when treated with* A. squamosa* [15]. These results further strongly substantiate the efficacy of* A. squamosa*in collagen maturation during wound repair. 

## 5. Conclusion

We have shown that the wound healing efficacy of* A. squamosa* in human dermal fibroblast by assessing cell proliferation and wound closure through scratch wound assay and the topical administration of ethanolic extract of* A. squamosa* promotes active synthesis of GAGs and collagen maturation during wound healing in normal and diabetic rats. This in turn is a positive sign of better wound healing.

## Figures and Tables

**Table 1:** Effect of *A.squamosa* on solubility pattern of collagen on day 8 wound tissues.

Group	Neutral salt soluble collagen	Acid soluble collagen	Pepsin soluble collagen	Insoluble collagen
Control	84.5 ± 10.4	590.5 ± 42.4	2289 ± 187.5	976.7 ± 10.2
Treated	97.8 ± 9.6	898.8 ± 86.5*	2812 ± 195.8*	1039.8 ± 17.4*
Diabetic control	64.9 ± 8.2	335.5 ± 66.1	1467 ± 173.4	841.3 ± 7.0
Diabetic treated	55.3 ± 9.2	465.0 ± 62.6*	1766 ± 90.4*	883.7 ± 13.2

Values (*μ*g/100 mg wet tissue) are expressed as mean ± SD (*n* = 6 animals).

*P < 0.05 is considered as significant compared to corresponding control.

**Table 2:** Effect of *A.squamosa* on susceptibility of insoluble collagen on day 8 wound tissues.

Group	2 M KCNS	6 M urea
Control	5.75 ± 0.79	6.70 ± 0.56
Treated	3.08 ± 0.58*	3.98 ± 0.63*
Diabetic control	3.49 ± 0.79	3.47 ± 0.79
Diabetic treated	2.33 ± 0.76*	2.29 ± 0.69*

Values (mg/100 mg collagen) are expressed as mean ± SD (*n* = 6 animals).

*P < 0.05is considered as significant compared to corresponding control.

**Table 3:** Aldehyde content of acid soluble collagen on day 8 wound tissues of control and *A.squamosa *treated rats.

Control	4.59 ± 1.01
Treated	8.51 ± 1.09*
Diabetic control	3.22 ± 0.90
Diabetic treated	5.20 ± 1.08*

Values (*μ*M/100 mg collagen) are expressed as mean ± SD (*n* = 6 animals).

*P < 0.05 is considered as significant compared to corresponding control.
